# SOCIAL SUPPORT AND LONELINESS AS DETERMINANTS OF THE ONSET OF DISABILITY AMONG PUERTO RICAN OLDER ADULTS

**DOI:** 10.1093/geroni/igac059.1432

**Published:** 2022-12-20

**Authors:** Amilcar Matos-Moreno, Neil Mehta, Eduardo Villamor, Lu Wang, Carlos Mendes de Leon

**Affiliations:** The Pennsylvania State University, Bayamon, Pennsylvania, Puerto Rico; UTMB, Galveston, Texas, United States; University of Michigan, Ann Arbor, Michigan, United States; University of Michigan, Ann Arbor, Michigan, United States; University of Michigan, Ann arbor, Michigan, United States

## Abstract

**Background:**

The effect of loneliness and social support on health is poorly understood among older Puerto Ricans. As family size continues to decrease in Puerto Rico due to out-migration, a higher number of older adults have fewer family members to rely on, which may lead to detrimental health outcomes.Method: Using both waves (2002-03 and 2006-07) of the Puerto Rican Elderly: Health Conditions database, we examined the association between social support, living alone, and incident disability among a sample of older adults over 60 years of age residing in Puerto Rico. Disability was defined as the occurrence of difficulties with Activities of Daily Living (ADLs).

**Results:**

13.4% of older adults in our sample developed some form of disability. Older adults who developed a disability indicated receiving higher levels of social support (2.04 vs. 1.64) and loneliness (30.7% vs. 22.8%). Using multivariate logistic regression, we found that receiving social support increased the odds of developing a disability by 17% (OR: 1.17; CI: 1.02 – 1.35). Older adults who live alone had 58% higher odds of developing a disability (OR: 1.58; CI: 1.01 – 2.46).

**Conclusion:**

The presence of social support and loneliness was correlated with a population prone to developing disabilities. Our findings concur with the well-established literature on psychosocial determinants in late life. However, this study represents the first attempt to understand psychosocial measures and disability in Puerto Rico. Public health organizations and healthcare systems must develop new societal mechanisms of support for older adults at risk of developing disabilities.

